# Maternal Vitamin D Deficiency and Its Effects on Pregnancy Outcomes

**DOI:** 10.1002/fsn3.71377

**Published:** 2026-01-26

**Authors:** Yakubu Ibrahim, Amilia Afzan Mohd Jamil, Norshariza Nordin, Su Peng Loh, Nurul Iftida Basri

**Affiliations:** ^1^ Department of Obstetrics and Gynaecology, Faculty of Medicine and Health Sciences Universiti Putra Malaysia Serdang Selangor Darul Ehsan Malaysia; ^2^ Department of Medical Laboratory Science, Faculty of Allied Health Sciences, College of Medical Sciences Ahmadu Bello University Zaria Kaduna State Nigeria; ^3^ Department of Biomedical Sciences, Faculty of Medicine and Health Sciences Universiti Putra Malaysia Serdang Selangor Darul Ehsan Malaysia; ^4^ Department of Nutrition and Dietetics, Faculty of Medicine and Health Sciences Universiti Putra Malaysia Serdang Selangor Darul Ehsan Malaysia

**Keywords:** cesarean section, gestational diabetes, gestational hypertension, Group B streptococcus, pregnancy outcomes, vitamin D deficiency

## Abstract

Vitamin D deficiency remains a global health concern for pregnant women. Adequate vitamin D is vital for optimal fetal development, immune regulation, and preventing adverse pregnancy outcomes. This study aims to determine the impact of vitamin D deficiency on pregnancy complications among a Malaysian cohort. This was a prospective observational study involving 414 pregnant women. Data was collected using a structured interviewer‐administered questionnaire, including sociodemographic and anthropometric characteristics, vitamin D risk factor assessment questions, vitamin D status, and follow up for pregnancy outcomes. Serum vitamin D level was determined using electrochemiluminescence immunoassay (ECLIA). The prevalence of vitamin D deficiency was 64.7%. Participants' daily median total vitamin D intake was 11.2 μg/day which was significantly low compared with the vitamin D recommended nutrient intake (RNI) of the study population. The study found a significant association between vitamin D status and pregnancy complications: gestational hypertensive disorder ( ꭓ^2^ = 9.024; *p* = 0.011), preterm birth (ꭓ^2^ = 8.249; *p* = 0.016), and Group B Streptococcus carrier (ꭓ^2^ = 7.379; *p* = 0.025). Participants who reported vitamin D consumptions during pregnancy had decreased likelihood of gestational hypertensive disorders (aOR = 0.278 (0.08–4.79) 95% CI; *p* = 0.0001), Group B Streptococcus carriage (aOR = 0.282 (0.08–0.99) 95% CI; *p* = 0.048), and decreased likelihood of cesarean section (aOR = 0.580 (95% CI: 0.347–0.967); *p* = 0.037). Vitamin D deficiency was significantly associated with increased risk of gestational hypertensive disorders, Group B Streptococcus carriage and cesarean section. It can be suggested that maternal vitamin D deficiency might be associated with an increased risk of some adverse pregnancy outcomes. Further interventional research is required to confirm a causal relationship, meanwhile promoting adequate vitamin D status among pregnant women may be beneficial.

## Graphical abstract

Vitamin D deficiency was highly prevalent among Malaysian pregnant women, with intake below recommended levels. Deficient vitamin D status was significantly associated with gestational hypertensive disorder, preterm birth, Group B Streptococcus carriage, and cesarean section. Vitamin D consumption during pregnancy showed a protective effect against hypertensive disorders, Group B Streptococcus infection, and cesarean section. It can be suggested that maternal vitamin D deficiency might be associated with an increased risk of some adverse pregnancy outcomes.

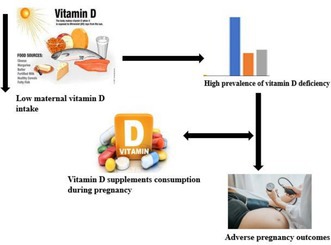

**Abbreviations**
2‐HPP: 2‐h post‐prandial
aOR: adjusted odd ratio
BMI: body mass index
BV: bacterial vaginosis
CI: confidence interval
FFQ: food frequency questionnaire
FPG: fasting plasma glucose
Hb: hemoglobin
HSAAS: Hospital Sultan Abdul Aziz Shah
IOM: Institute of Medicine
IQR: interquartile range
LMP: last menstrual period
OGTT: oral glucose tolerance test
PTB: preterm birth
RNI: recommended nutritional intake
RPM: revolution per minute
SD: standard deviation
USDA: United States Department of Agriculture

## 1 Introduction

Maternal hypovitaminosis D has received attention in the field of obstetrics due to its possible negative impact on pregnancy (van der Pligt et al. 2018). The prevalence of vitamin D deficiency is on the rise with varying prevalence rates across countries (Fareed 2019; Hossain et al. 2011; Tabrizi et al. 2018). Pregnant mothers in Asia, Africa, the Gulf region, and Latin are thought to be much more vulnerable to vitamin D deficiency with the highest prevalence rates worldwide between 60% and 90% (Bonevski et al. 2013; Davoudi‐Kiakalayeh et al. 2017; Prentice et al. 2009; van der Pligt et al. 2018; Van Schoor and Lips 2011). Malaysia, like few other countries situated on the equator, receives ample sunlight throughout the year. Despite this, research has revealed that vitamin D deficiency is common among Malaysian pregnant women and was thought to be associated with pregnancy complications (Chasan‐taber et al. 2004; de Almeida Mens et al. 2018; Jamil et al. 2019; Syed Nor et al. 2022). Previous studies reported that the prevalence of vitamin D deficiency was 90.4% among first trimester women, 42.6% during the second trimester, and 50.2% during the third trimester (Palacios and Gonzalez 2014; Pilz et al. 2018; World Health Organisation 2005 2005). According to the World Health Organization (WHO), several population‐based studies have linked low vitamin D status to certain risk factors that affect vitamin D levels (World Health Organization 2005 2005). For instance, low vitamin D nutritional intake, limited sunshine exposure, skin pigmentation, obesity, and certain medical conditions, cultural practices, and clothing choices due to religious rites have effects on vitamin D status (Campbell et al. 2023; Fogacci, Fogacci, Banach, et al. 2020; Jamil et al. 2019; Palacios and Gonzalez 2014; Prentice et al. 2009). Vitamin D dietary intake or use of vitamin D supplements during pregnancy is one of the important factors that contribute to vitamin D status; however, there is no policy in place to support its implementation in the study area (Pilz et al. 2018). Dietary vitamin D supplementation is only legislated in certain countries (Campbell et al. 2023; Fogacci, Fogacci, and Cicero 2020; Ku et al. 2024; Lee et al. 2021), while in others, there is no law regulating the fortification of vitamin D in food, including Malaysia (Al‐Shaikh et al. 2016; Al Khalifah et al. 2020; Hussein et al. 2018).

Vitamin D deficiency during pregnancy can lead to a myriad of complications during pregnancy and delivery (Alanazi et al. 2022). Emerging evidence revealed that maternal hypovitaminosis D is associated with various prenatal and neonatal complications such as gestational hypertensive disorders (GHD), gestational diabetes mellitus (GDM), preterm birth (PTB), bacterial vaginosis (BV), and increased rates of cesarean section (CS) (Bodnar et al. 2014; Caccamo et al. 2020; Farajian‐Mashhadi et al. 2020; Kalok et al. 2020; Liu et al. 2020; Monier et al. 2019; Phoswa and Khaliq 2021; Rajput et al. 2013). Even though maternal hypovitaminosis D is a global issue, there is a paucity of studies exploring the association between vitamin D levels and adverse pregnancy outcomes. Therefore, a thorough understanding of the existing problem is essential to recognize the role of maternal vitamin D deficiency in pregnancy outcomes. The present study aims to determine the impact of maternal vitamin D deficiency on pregnancy outcomes among Malaysian pregnant women.

## 2 Materials and Methods

The study was conducted at Hospital Sultan Abdul Aziz Shah (HSAAS), Universiti Putra Malaysia (UPM). HSAAS is a major tertiary healthcare center and serves as the teaching hospital for UPM. The cross‐sectional prospective observational study was conducted between July 5, 2022 and July 4, 2024. Pregnant women receiving antenatal care at the Department of Obstetrics and Gynaecology, who met the inclusion and exclusion criteria, were recruited using purposive sampling method. The study included women with a singleton pregnancy in the second trimester and beyond, viable pregnancy at recruitment, agreement to participate with signed written informed consent, and willingness to follow up for prenatal and obstetric outcomes assessment. On the other hand, women with pre‐existing chronic diseases, conditions affecting vitamin D metabolism, or those unwilling to consent or follow up were excluded from the study.

### 2.1 Definitions of Pregnancy Outcomes

Pregnancy outcomes being analyzed were GHD, GDM, PTB, Group B Streptococcus (GBS) carriage, and mode of delivery. For this study, GHD includes gestational hypertension and preeclampsia. Gestational hypertension was diagnosed when a pregnant individual develops new‐onset hypertension (systolic blood pressure ≥ 140 mmHg or diastolic blood pressure ≥ 90 mmHg) after 20 weeks of gestation without evidence of proteinuria or systemic organ dysfunction (American College of Obstetrics and Gynaecology 2020). Preeclampsia is diagnosed when hypertension is accompanied by significant proteinuria of ≥ 300 mg in 24‐h urine, a protein‐to‐creatinine ratio ≥ 0.3, or signs of end‐organ damage, such as aberrant renal, hepatic, neurological, or hematological dysfunctions (American College of Obstetrics and Gynaecology 2020).

GDM was diagnosed based on the Malaysian Clinical Practice Guidelines (Guideline 2017). Screening was made at booking for women with risk factors and at 24–28 weeks for women aged ≥ 25 without additional risk factors. Diagnosis was made using a 75 g oral glucose tolerance test (OGTT) and threshold values of fasting plasma glucose (FPG) ≥ 5.1 mmol/L or 2 h post‐prandial (2‐HPP) ≥ 7.8 mmol/L. PTB was considered as the delivery of a live infant before 37 weeks of gestation, calculated based on the first day of the last menstrual period (LMP) or revised estimated due date obtained from dating ultrasound scan.

Anemia was diagnosed when the hemoglobin (Hb) is less than 10.5 g/dL in the second and third trimester (Anemia in Pregnancy: ACOG Practice Bulletin, Number 233 2021). As the hospital practices universal screening of GBS, all participants were screened for GBS between 35 and 38 weeks, or earlier if they present with symptoms suggestive of preterm labor. Mode of delivery were divided into vaginal delivery, assisted vaginal delivery (both ventouse, and forceps delivery) and cesarean section.

### 2.2 Sample Size Determination

The minimum required sample size for this study was determined using the single‐population proportion formula as described by Charan and Biswas (2013).

$$n=\frac{Z^{2}\mathrm{pq}}{d^{2}}$$

where: *n* = estimated sample size, *Z* = level of significance at 95% confidence interval (1.96), using a standard prevalence *p* = 0.5, *q* = 1 − *p* = 0.5, *d* = tolerable margin of error (5%) = 0.05.

$$n=\frac{Z^{2}\mathrm{pq}}{d^{2}}=\frac{\left(1.96\right)\;\left(1.96\right)\times 0.5\times 0.5}{\left(0.05\right)\;\left(0.05\right)}\approx \frac{385\times 0.25}{0.0025}\approx 385$$

The calculated sample size was adjusted to account for potential nonresponse and incomplete data. Adjustment is consistent with established recommendations in survey methodology literature, which advocate a 5%–10% (Charan and Biswas 2013; Naing et al. 2022). An attrition rate of 5% was incorporated into the minimum required sample size (Hussein et al. 2024).

The minimum required sample size $\mathrm{nm}=\frac{n_{0}}{1-r}=\frac{385}{1-0.07}=\frac{385}{0.93}=403$

Sample size required for this study is 403.

### 2.3 Data Collection

#### 2.3.1 Anthropometric Measurements

The weight of the women was measured approximately 0.1 kg using Pearson's digital scale with the woman standing without shoes and in lightweight clothing. Pre‐pregnancy weight was obtained from mothers' antenatal booking cards. The measurement of height was to the nearest 0.5 cm with the subject standing by the stadiometer without shoes and the reading was taken at the level of the vault of the skull. The pre‐pregnancy body mass index (BMI) was calculated using pre‐pregnancy weight and height using the formula:

$\mathrm{BMI}\;\left(\mathrm{kg}/m^{2}\right)=\text{weight}\;\left(\mathrm{kg}\right)/\text{height}\left(m^{2}\right)$.

#### 2.3.2 Vitamin D Dietary Intake

The validated food frequency questionnaire (FFQ) for vitamin D was utilized to determine the amount of vitamin D consumed in diets (File S1; Isa et al. 2022). Participants' consumption of vitamin D foods such as fish, milk, poultry and related products, mushrooms, commercial vitamin D fortified foods such as milk and dairy products, beverages (biscuits, snacks, confectionery, and savory snack foods) and dietary supplements use during pregnancy were recorded. Participants were asked to recall the food they had consumed during the previous month, including the brand (for commercial foods), food portion size and frequency of intake. They were also asked about the amount, frequency of intake, and type of vitamin D supplements they had taken from the previous month. The vitamin D content of naturally vitamin D‐rich foods was obtained from the United States Department of Agriculture (USDA), National Nutrient Database for Standards, Agricultural Research Service (2018) and the “Singapore Energy and Nutrient Composition of Food Database (Health Promotion Board, Singapore).” Other information on vitamin D content of the supplements was obtained from the product labels and company websites.

#### 2.3.3 Daily Vitamin D Intake Calculation

Vitamin D content of foods (or supplements), multiplied by the dosage or portion size and frequency of consumption was used to determine the subject's daily vitamin D intake. The vitamin D intake from different food groups was added to obtain the total vitamin D intake from food sources and supplements. Subject's vitamin D adequacy was assessed by comparing the daily median total vitamin D intake with vitamin D RNI (15 μg/day) for Malaysian pregnant women (IOM 2011; MOH Malaysia 2017).

### 2.4 Blood Sample Collection, Processing, and Storage

About 6.0 mL of venous blood was collected from each participant into the plain vacutainer blood bottle. The blood was allowed to sufficiently clot before it was transported to the laboratory under cold chain. It was centrifuged at a speed of 3000 rpm for 10 min and the clear non‐hemolyzed serum was harvested and aliquoted into cryovials and kept at −20°C prior to vitamin D analysis.

### 2.5 Laboratory Determination of 25 (OH)D_3_

Serum 25‐hydroxycholecalciferol (25(OH)D_3_), a marker used to assess vitamin D status, was determined using the electrochemiluminescence immunoassay (ECLIA) technique using the Cobas e411 analyser (Roche, Germany). Vitamin D status was classified according to the Institute of Medicine (IOM) guideline: Normal = > 50 nmol/L; Insufficient = 30–50 nmol/L; Deficient < 30 nmol/L.

### 2.6 Statistical Analysis

Data analysis was conducted using the IBM SPSS v27.0 statistical software. A normality test was performed on the data. Normally distributed continuous variables were presented as mean (SD) while non‐normally distributed variables were presented as median (IQR). Categorical variables were analyzed using Pearson's chi‐square test. A non‐parametric test was used for non‐normally distributed data; the Wilcoxon signed‐rank test was used to compare the median total vitamin D dietary intake with the vitamin D RNI of the study population. Binary logistic regression analysis was used to predict the association between continuous covariates and categorical predictors with the outcome variable (pregnancy complications and mode of delivery). The level of significance was considered at *p* ≤ 0.05 at a 95% confidence interval (CI).

## 3 Results

A total of 425 patients were recruited for this study; however, 11 participants dropped out, leaving 414 participants. Background characteristics of the study participants were presented in Table 1. The mean age of participants was 32.4 ± 4.5 years, with 64.3% aged between 24 and 34 years. Most participants, 54.7% hold a degree, and 95.7% were of Malay descent. Income distribution showed 45.4% were low‐income earners, middle‐income (46.1%), and high‐income (8.5%). Approximately, 88% were working mothers. The majority of participants (71.0%) were in their third trimester of pregnancy, with 68% being multiparous and 32% primiparous. The pre‐pregnancy mean weight was 64.7 ± 14.1 kg, with 44% having a normal BMI, 29% overweight, and 23.9% obese. The mean systolic blood pressure at booking was 114.9 ± 14.6 mmHg, and diastolic pressure was 74.6 ± 9.1 mmHg. 19% of participants have a family history of both GHD and GDM, 32% GHD alone, while 19% GDM. About 9% women had a prior history of GDM, GHD (3%), while 15% had a previous history of CS and PTB (1%). Most subjects (96%) wore veiled dress.

**TABLE 1:** Distribution of the sociodemographic and clinical characteristics (*n* = 414).

Variables	*N* (%)	Mean
	*n* = 414	
Age		
18–24	6 (1.4)	32.4 (4.5)
25–34	266 (64.3)	
≥ 35	142 (34.3)	
Level of education		
Secondary and below	41 (9.9)	
Certificate and diploma	147 (35.4)	
Degree and above	227 (54.7)	
Ethnicity		
Non‐Malay (Chinese, Indian, others)	18 (4.30)	
Malay	396 (95.7)	
Work status		
Employed	364 (87.9)	
Unemployed	50 (12.1)	
Household income/month		
Low (< 4850)	188 (45.4)	
Middle (RM 4851–RM 10970)	191 (46.1)	
High (> RM 10,971)	35 (8.5)	
Gestation age at recruitment		
Second trimester	120 (29.0)	
Third trimester	294 (71.0)	
Gestation age at delivery		
Term delivery (≥ 37 weeks)	374 (90.3)	
Preterm delivery (< 37 weeks)	40 (9.7)	
Parity		
Nulliparous	133 (32.1)	
Multiparous	281 (67.9)	
Pre‐pregnancy BMI (kg/m^2^)		
Under weight (< 18)	13 (3.1)	26.2 (5.4)
Normal (18.5–24.9)	180 (43.5)	
Overweight (25–29.9)	122 (29.5)	
Obese (≥ 30)	99 (23.9)	
Blood pressure (mmHg)		
Systolic blood pressure		114.9 (14.6)
Diastolic blood pressure		74.6 (9.1)
Family history		
None	114 (30.2)	
GHD and GDM	79 (19.1)	
GHD	131 (31.6)	
GDM	79 (19.1)	
Previous obstetric history		
No complication	236 (71.3)	
GHD	13 (3.1)	
GDM	39 (9.4)	
PTB	4 (1.0)	
CS	63 (15.2)	
Use of vitamin D supplements		
Yes	89 (21.5)	
No	325 (78.5)	
Type of dress		
Non‐veiled	15 (3.6)	
Veiled	399 (96.4)	

*Note:* BMI using WHO classification.

Abbreviations: CS, cesarean section; GDM, gestational diabetes mellitus; GHD, gestational hypertensive disorder; PTB, preterm birth.

**FIGURE 1:**
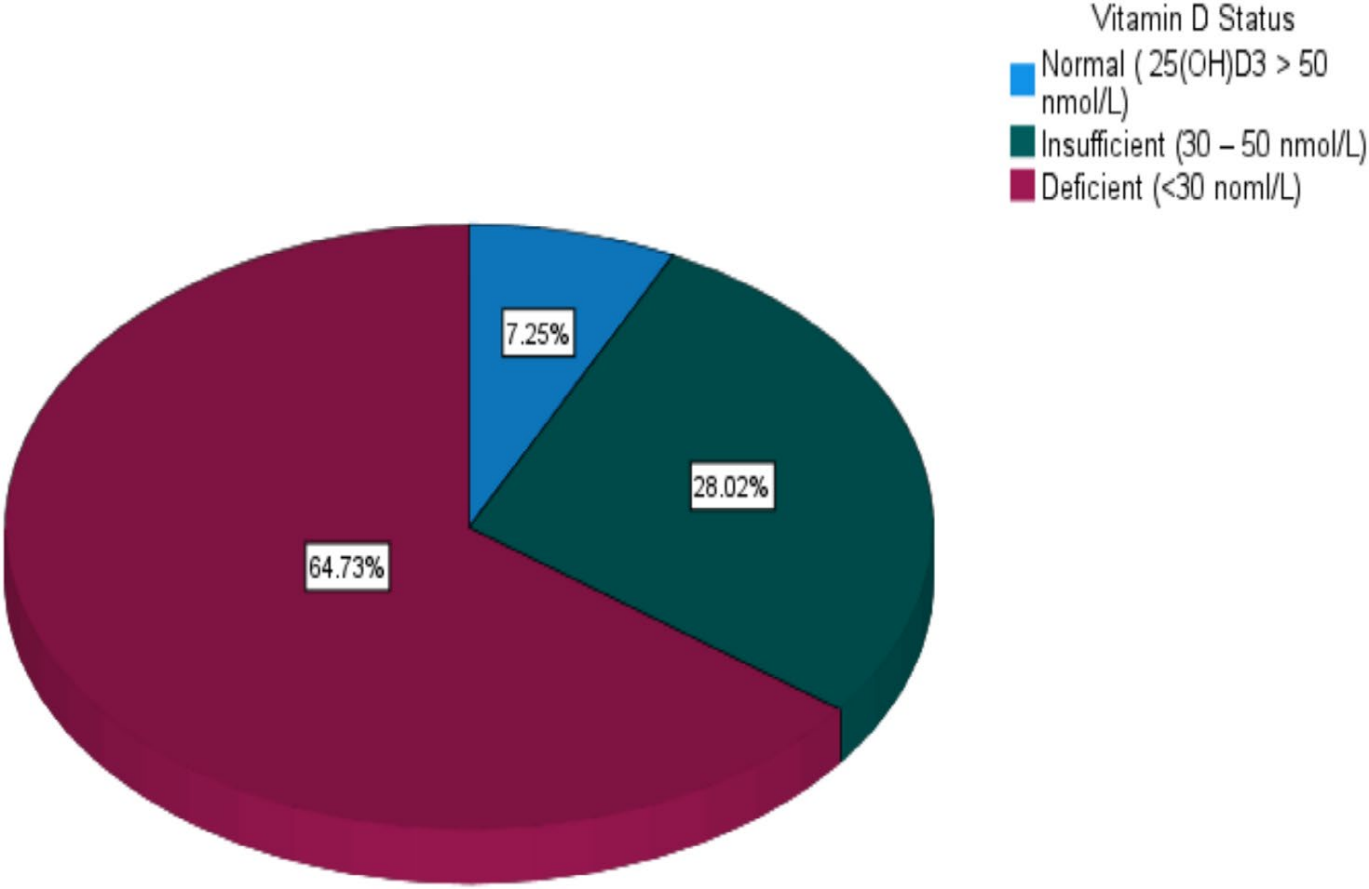
Distribution of vitamin D status among the study participants. Classification of vitamin D status was according to the Institute of Medicine guideline. Normal = > 50 nmol/L; Insufficient = 30–50 nmol/L; Deficient < 30 nmol/L.

Table 2 shows pregnancy outcomes among the study participants; the proportion of participants with GDM was 24%, GHD (22%), PTB (10%), anemia (7.2%), and GBS positive (9%). About 42% of participants were delivered via CS, normal delivery (41%), and assisted vaginal delivery (17%). Figure 1 shows the distribution of vitamin D status among the study participants and was classified according to the IOM guideline. Vitamin D deficiency was found in 64.7% of study participants, 28% had insufficient levels, and only 7.2% had normal levels.

**TABLE 2:** Proportion of pregnancy outcomes among the study participants.

Pregnancy outcomes	*N*	%
	*n* = 414	
GHD		
No	323	78.0
Yes	91	22.0
GDM		
No	314	75.8
Yes	91	24.2
PTB		
No	374	90.3
Yes	40	9.7
Anemia		
No	384	92.8
Yes	30	7.2
GBS carriage		
No	376	90.8
Yes	38	9.2
Mode of delivery		
Normal delivery	169	40.8
Assisted vaginal delivery	72	17.4
Cesarean section	173	41.8

Abbreviations: GDM, gestational diabetes mellitus; GHD, gestational hypertensive disorder; PTB, preterm birth.

The study evaluated the participants' vitamin D dietary intake from various food sources and supplements intake during pregnancy. The median daily vitamin D intake was highest for dairy products (114 g/day), followed by fish (47.2 g/day), beverages and cereals (21.0 g/day), chicken and eggs (20.0 g/day). The participants' median daily vitamin D intake from food was 4.8 μg/day, and the mean intake from supplements was 2.6 ± 5.8 μg/day. The overall total daily vitamin D intake was 11.2 μg/day. The Wilcoxon signed‐rank test was used to compare the participants' total daily vitamin D intake with the population RNI (15.0 μg/day). There was a significant difference (*p* = 0.016) between the participants' median daily vitamin D intake and vitamin D RNI for Malaysian pregnant women, as shown in Table 3.

**TABLE 3:** Distribution of vitamin D dietary and supplement intake and recommended nutrition intake (RNI).

Variables	Median (Q1 − Q3)/Mean (SD)	*p*
Vitamin D dietary and supplements intake and daily recommended nutrition intake (RNI)		
Fish and sea related foods (g/day)	47.2 (0.0–47.2)	
Dairy products and related foods (g/day)	114.0 (0.0–120.0)	
Beverages and cereals related (g/day)	21.0 (21.0–42.0)	
Chicken, eggs and poultry related foods (g/day)	20.0 (6.1–61.8)	
Total intake from food (μg/day)	4.8 (3.1–7.5)	
Total intake from supplements (μg/day)	2.6 (5.8)	
Total intake (foods and supplements) (μg/day)	11.2 (6.8–20.6)	0.016*

*Note:* Total vitamin D intake = Intake from foods + intake from supplements; vitamin D recommended nutrition intake for Malaysian pregnant mothers = 15 μg/day; Wilcoxon signed‐rank test was used to compare the median total vitamin D dietary intake with the vitamin D RNI.

* *p* ≤ 0.05.

Analysis of the association between vitamin D status with pregnancy outcomes found that there was a significant association between vitamin D status and GHD (ꭓ^2^ = 9.024; *p* = 0.011), PTB (ꭓ^2^ = 8.249; *p* = 0.016), and GBS carriage (ꭓ^2^ = 7.379; *p* = 0.025). Similarly, there was a significant association between vitamin D status and mode of delivery (ꭓ^2^ = 11.427; *p* = 0.002) among the study participants, as shown in Table 4.

**TABLE 4:** Association of different levels of vitamin D status with pregnancy outcome parameters.

Pregnancy outcomes	Vitamin D status (*n* = 414)				
	Normal *N* (%)	Insufficient *N* (%)	Deficient *N* (%)	ꭓ^2^	*p*
GHD					
No	26 (86.7)	100 (86.2)	197 (73.5)	9.024	0.011*
Yes	4 (13.3)	16 (13.8)	71 (26.5)		
GDM					
No	22 (73.3)	88 (75.9)	204 (76.1)	0.114	0.944
Yes	8 (26.7)	28 (24.1)	64 (23.9)		
PTB					
No	28 (93.3)	112 (96.6)	234 (87.3)	8.249	0.016*
Yes	2 (6.7)	4 (3.4)	34 (12.7)		
Anemia					
No	28 (93.3)	109 (94.0)	247 (92.2)	0.407	0.816
Yes	2 (6.7)	7 (6.0)	21 (7.8)		
GBS carriage					
No	26 (86.7)	99 (85.3)	251 (93.7)	7.379	0.025*
Yes	4 (13.3)	17 (14.7)	17 (6.3)		
Mode of delivery					
Normal delivery	13 (6.6)	71 (36.2)	112 (57.1)	11.427	0.022*
Assisted vaginal delivery	6 (8.3)	14 (19.4)	52 (72.2)		
Cesarean section	7 (4.8)	35 (24.0)	104 (71.2)		

*Note:* Vitamin D status; Normal = > 50 nmol/L; Insufficient = 30–50 nmol/L; Deficient < 30 nmol/L. *denotes To provide references for normal, insufficient and deficient level.

Table 5 shows that participants who consumed vitamin D supplements during pregnancy were significantly less likely to develop GHD (aOR = 0.278 (0.08–4.79) 95% CI; *p* = 0.0001) and significantly protected against GBS carriage (aOR = 0.282 (0.08–0.99) 95% CI; *p* = 0.048). On the other hand, participants between the ages of 25 and 34 years were nearly 11 times more likely to have PTB as compared to participants in other age groups (aOR = 10.979 (1.702–70.839) 95% CI; *p* = 0.012). Additionally, it was found that vitamin D deficient participants had 2.672 odds of GBS carriage as compared to participants with normal vitamin D status (aOR = 2.672 (1.278–5.584) 95% CI; *p* = 0.009).

**TABLE 5:** Association between risk factors of vitamin D deficiency and pregnancy outcomes.

Covariates	GHD		GDM		PTB		Anemia		GBS carriage	
	aOR (95% CI)	*p*	aOR (95% CI)	*p*	aOR (95% CI)	*p*	aOR (95% CI)	*p*	aOR (95% CI)	*p*
25 (OH)D_3_ (nmol/L)	0.985 (0.934–1.03)	0.533	0.973 (0.93–1.02)	0.220	0.995 (0.93–1.07)	0.880	1.009 (0.94–1.08)	0.804	—	—
Total vitamin D intake (μg/day)	1.001 (0.99–1.01)	0.579	0.998 (0.99–1.00)	0.524	0.990 (0.96–1.02)	0.489	0.990 (0.96–1.02)	0.544	0.993 (0.97–1.02)	0.628
Vitamin D status										
Normal	Reference		Reference		Reference		Reference		Reference	
Insufficient	0.623 (0.08–4.79)	0.650	2.682 (0.45–15.96)	0.278	0.483 (0.03–9.34)	0.630	0.472 (0.02–10.25)	0.633	2.296 (0.69–7.63)	0.175
Deficient	0.529 (0.21–1.36)	0.187	1.596 (0.71–3.60)	0.261	0.292 (0.067–1.28)	0.103	0.679 (0.17–2.74)	0.586	2.672 (1.278–5.584)	0.009*
Use of vitamin D supplements										
No	Reference		Reference		Reference		Reference		Reference	
Yes	0.278 (0.13–0.59)	0.0001**	0.827 (0.46–1.49)	0.529	0.634 (0.23–1.72)	0.370	0.990 (0.96–1.02)	0.544	0.282 (0.08–0.99)	0.048*
Type of dressing										
Non‐veiled	Reference		Reference		Reference		Reference		Reference	
Veiled	14.809 (0.66–333.76)	0.090	5.157 (0.51–52.46)	0.166	12.318 (0.45–335.99)	0.137	2.005 (0.06–69.55)	0.701	5.179 (0.399–67.42)	0.209
Age (years)										
18–24	Reference		Reference		Reference		Reference		Reference	
25–34	1.795 (0.27–11.82)	0.543	3.067 (0.56–16.76)	0.196	10.979 (1.702–70.839)	0.012*	0.001 (0.001–0.002)	0.999	0.001 (0.01–0.02)	0.999
≥ 35	0.870 (0.51–1.50)	0.616	1.113 (0.66–1.87)	0.686	1.821 (0.778–4.261)	0.167	1.066 (0.452–2.514)	0.884	1.372 (0.62–3.04)	0.437
Level of education										
Secondary and below	Reference		Reference		Reference		Reference		Reference	
Certificate and diploma	0.599 (0.20–1.80)	0.361	1.905 (0.81–4.47)	0.138	1.417 (0.429–4.684)	0.567	1.534 (0.349–6.751)	0.571	0.563 (0.14–2.33)	0.428
Degree and above	1.384 (0.80–2.39)	0.245	1.255 (0.73–2.14)	0.407	1.021 (0.459–2.270)	0.959	2.118 (0.888–5.051)	0.091	0.686 (0.31–1.53)	0.358
Ethnicity										
Non‐Malay (Chinese, Indian, others)	Reference		Reference		Reference	0.250	Reference		Reference	
Malay	0.034 (0.01–1.02)	0.052	0.223 (0.02–2.32)	0.210	0.146 (0.04–5.06)	0.287	0.391 (0.01–12.32)	0.594	0.710 (0.06–8.54)	0.787
Household income										
Low (< 4850)	Reference		Reference		Reference		Reference		Reference	
Middle (RM 4851–RM 10,970)	1.016 (0.37–2.82)	0.975	0.491 (0.20–1.22)	0.124	1.139 (0.28–4.71)	0.857	0.485 (0.12–1.928)	0.304	2.368 (0.45–12.38)	0.307
High (> RM 10,971)	1.576 (0.62–4.02)	0.341	0.704 (0.31–1.59)	0.400	0.714 (0.18–2.83)	0.631	0.527 (0.15–1.85)	0.318	2.181 (0.46–10.33)	0.326

*Note:* Level of significance, **p < 0.0001, *p ≤ 0.05, at 95% confidence interval (CI).

Abbreviations: GBS, Group B streptococcus, GDM, gestational diabetes; GH, gestational hypertension.

Table 6 showed that participants with a moderate (certificate and diploma) level of education were 3.264 more likely to have assisted vaginal delivery as compared with participants with low (secondary and below) level of education (aOR = 3.264 (1.372–7.765) 95% CI; *p* = 0.007). High‐income earning participants were 4.942 more likely to deliver through assisted vaginal delivery as compared with low‐income earning participants (aOR = 4.942 (1.099–22.219) 95% CI; *p* = 0.037). Additionally, we found that participants who consume vitamin D supplements during pregnancy were 48% less likely to have CS as compared to those not consuming vitamin D supplements during pregnancy (aOR = 0.580 (0.347–0.967) 95% CI; *p* = 0.037).

**TABLE 6:** Association between risk factors of vitamin D deficiency and mode of delivery.

Factors/Covariates	Assisted vaginal delivery		Cesarean section	
	aOR (95% CI)	*p*	aOR (95% CI)	*p*
Daily total vitamin D intake (μg/day)	1.002 (0.999–1.005)	0.231	1.001 (0.998–1.004)	0.421
Vitamin D status				
Normal	Reference		Reference	
Insufficient	0.950 (0.353–2.562)	0.920	0.517 (0.228–1.171)	0.114
Deficient	0.587 (0.304–1.133)	0.112	0.677 (0.428–1.071)	0.096
Use of vitamin D supplements				
No	Reference		Reference	
Yes	0.804 (0.408–1.581)	0.527	0.580 (0.347–0.967)	0.037*
Type of dressing				
Non‐veiled	Reference		Reference	
Veiled	3.314 (0.489–22.474)	0.220	1.287 (0.177–9.363)	0.803
Age (years)				
18–24	Reference		Reference	
25–34	1.247 (0.195–7.985)	0.816	3.015 (0.507–17.916)	0.225
≥ 35	0.808 (0.453–1.442)	0.470	1.190 (0.754–1.879)	0.455
Level of education				
Secondary and below	Reference		Reference	
Certificate and diploma	3.264 (1.372–7.765)	0.007*	1.043 (0.484–2.247)	0.914
Degree and above	0.977 (0.519–1.841)	0.943	1.044 (0.658–1.658)	0.854
Ethnicity				
Non‐Malay (Chinese, Indian, others)	Reference		Reference	
Malay	0.938 (0.148–5.968)	0.946	0.294 (0.044–1.963)	0.207
Household income				
Low (< 4850)	Reference		Reference	
Middle (RM 4851–RM 10970)	2.820 (0.579–13.727)	0.199	0.519 (0.226–1.187)	0.120
High (> RM 10,971)	4.942 (1.099–22.219)	0.037*	0.509 (0.236–1.095)	0.084

*Note:* Binary logistic regression analysis, level of significance, **p < 0.0001, *p ≤ 0.05, at 95% confidence interval (CI).

## 4 Discussion

The present study found a significantly high prevalence of vitamin D deficiency (64.7%). The high prevalence found in this study was similar to other previous studies in Malaysia which reported varying prevalence of vitamin D deficiency between 42% and 91% (Bukhary et al. 2016; Ibrahim et al. 2025; Jamil et al. 2019; Woon et al. 2019). Other studies conducted in tropical nations have also reported high prevalence similar to this study (Annie Judkins 2006; Hossain et al. 2011; Roth et al. 2018; Tabrizi et al. 2018; Yang et al. 2021). For instance, in Western Indonesia, pregnant women in the third trimester had a prevalence of 61.3% (Aji et al. 2022) and 60.0% (Hanieh et al. 2014). In contrast, slightly higher prevalence of 75.5% was observed in Thailand (Pratumvinit et al. 2015). The reason for the varying prevalence across the studies could be due to the different methods of 25(OH)D analysis or different cut‐off levels. Maternal vitamin D deficiency in sunny countries of Southeast Asia and the Middle East was higher than those reported in Western countries (Zhao et al. 2017). This could be due to factors such as skin color, cultural and religious practices, and legislation on vitamin D fortification of foods (Prentice et al. 2009).

Vitamin D intake is one of the modifiable risk factors of vitamin D deficiency. The present study found that the median daily total vitamin D intake was 11.2 μg/day. This was significantly lower compared to the vitamin D RNI of the study population and the Institute of Medicine (IOM) recommended intake levels of 15 μg/day for pregnant women. The finding aligns closely with the finding of previous work, which found the average total vitamin D intake of 11.5 μg/day (Yong et al. 2017) and our finding was slightly higher than 10.2 μg/day previously reported in the study area (Woon et al. 2019). Interestingly, our reported median total intake was higher than that reported in Indonesia (7.9 μg/day; Aji et al. 2022; Oktaria et al. 2020), Japan (5.5 μg/day; Miyake et al. 2014), and the Netherlands (5.9 μg/day; Looman et al. 2019), Sweden (9.3 μg/day; Brembeck et al. 2013). These could be attributed to several factors, including lack of knowledge about vitamin D‐rich foods, socioeconomic disparities that limit access to fortified foods and dietary supplements, as well as population genetic variability on vitamin D response. In contrast, Canadian studies reported higher (17.2 and 21.8 μg/day) intakes than that found in the present study (Aghajafari et al. 2013; Savard et al. 2018). Their better intake levels were largely attributable to the widespread use of vitamin D supplements among the study participants. Prenatal vitamin D supplement consumption contributes to enhanced vitamin D levels, although their availability and usage are often driven by individual requirements and policy‐driven initiatives. It is noteworthy that the distribution of prenatal multivitamins is uncommon but is based on availability and individual requirements (Lee et al. 2021).

The lower vitamin D intake among our study participants could support the need for targeted interventions to address vitamin D deficiency. Comprehensive studies assessing dietary vitamin D intake are essential to generate evidence for policy formulation. Promoting vitamin D supplementation for at‐risk groups including pregnant women, enhancing healthcare professionals' awareness, and fortified food policies are important strategies to address maternal vitamin D deficiency.

This study found that participants who consume vitamin D supplements during pregnancy were less likely to develop GHD as compared to participants not consuming vitamin D supplements (aOR = 0.278 (0.08–4.79) 95% CI; *p* = 0.0001). This is in line with the findings of the previous study who reported that pregnant women on vitamin D supplements before 20 weeks of gestation seem to provide protective effects against preeclampsia (Fogacci, Fogacci, Banach, et al. 2020), and hypertension (Burris et al. 2014; Dkhar et al. 2019; Haugen et al. 2009; Hollis and Wagner 2004). Previous case–control studies also reported that pregnant women with low 25 (OH)D_3_ levels had an increased risk of developing preeclampsia (Abedi et al. 2014; Ullah et al. 2013). Vitamin D is believed to support vascular health by regulating calcium homeostasis, lowering blood pressure, suppressing renin biosynthesis, and modulating the renin‐angiotensin system, which plays a crucial role in blood pressure regulation (Chi et al. 2022). Adequate vitamin D intake may also positively influence adipokine production, contributing to improved endothelial function and overall vascular health (Rezende et al. 2012).

Additionally, participants with vitamin D deficiency have 2.672 odds of carrying GBS bacteria as compared to participants with normal vitamin D level. Invasive GBS infections during pregnancy have been reported to be a major cause of adverse pregnancy outcomes, including PTB (Guevara et al. 2020; Patras and Nizet 2018; Russell et al. 2017). Several factors can influence vaginal colonization and invasive infection leading to PTB. A meta‐analysis from over 10,000 pregnant women found that maternal vitamin D deficiency is associated with 1.29 increase odds of having PTB (Qin et al. 2016). Similarly, another meta‐analysis also found that vitamin D supplementation during pregnancy may reduce the rates of PTB (Ravel et al. 2011). Maternal vitamin D deficiency may increase the risk of GBS colonization through impaired immune regulation, reduced antimicrobial peptide production, and disruption of the vaginal microbiome (Guevara et al. 2020). These alterations facilitate GBS overgrowth and ascending infection, triggering inflammation that could lead to PTB (Mendz 2023). The observed protective effect on participants who reported vitamin D supplements use against GBS supports its immunomodulatory and anti‐inflammatory role and in maintaining genital tract health.

CS is associated with longer maternal recovery, higher surgical risks, anesthetic side effects, future pregnancy complications, and potential emotional impacts, with associated economic, birth and family plan effects (Sandall et al. 2018). Our findings revealed that participants who reported vitamin D supplements consumption had a lower risk of CS. A previous study by Hubeish et al. (2018) found that the rates of CS due to failure to progress in labor and failed induction were three times more frequent among vitamin D deficient (≤ 70 nmol/L) participants than in controls (Hubeish et al. 2018). Additionally, it has been suggested that maternal vitamin D deficiency has a substantial effect on postpartum bleeding due to a higher likelihood of uterine atony (Kalok et al. 2020). Insufficient vitamin D levels could lead to hypocalcemia, which impairs uterine muscle contraction, resulting in uterine atony (Sörsjö Stevenazzi et al. 2024). This condition can hinder labor progression due to uncoordinated uterine contraction, increasing the likelihood of CS. In contrast, a previously reported study posited non‐association between vitamin D deficiency and risk of CS (Gernand et al. 2016). Variability in population demographics, dietary habits, and baseline vitamin D levels could influence the delivery outcomes. Additionally, genetic factors, such as polymorphisms in the vitamin D receptor gene, may affect maternal vitamin D and calcium metabolism, potentially affecting labor progression. The findings of a multi‐ethnic study observed an increased risk of CS in Chinese and Indian women but not in Malay women (Loy et al. 2015). This highlighted the potential ethnic and genetic variability in the response to vitamin D. Additionally, different cultural practices, such as diet, sun exposure, and supplementation habits, might also contribute to these disparities. Future studies into the genetic, epigenetic, and environmental factors are essential to clarify these variations.

### 4.1 Strengths and Limitations

The strengths of our study include its significant contribution in understanding the impact of maternal vitamin D deficiency on pregnancy outcomes. The findings provide a robust platform for efforts to reduce pregnancy and childbirth complications associated with inadequate vitamin D levels. Importantly, the study highlights the role of vitamin D dietary intake as a modifiable risk factor, emphasizing the need to assess and address dietary intake as part of comprehensive maternal health initiatives. These findings could be used to support the need for periodic revision of population‐specific pregnancy vitamin D RNI and guide strategies to address maternal vitamin D deficiency. The prospective design of this study and substantial sample size also enhance the reliability and generalizability of our findings.

As participants were required to recall their vitamin D dietary and food consumption for the past month, this may result in some recall bias. Majority of our participants were Malay; hence, the results may not be generalized to other populations due to the dietary and lifestyle disparities as well as influence of genetic variability. Additionally, vitamin D levels were measured only once, which might not represent levels throughout pregnancy. Other than that, we do not have detailed information on the participant's sunlight exposure. Future studies should take into cognizance the setbacks of the present study.

## 5 Conclusions

The study found a higher prevalence of maternal vitamin D deficiency and associated modifiable risk factors. It was suggested that vitamin D deficiency might be associated with several adverse pregnancy outcomes. These results encourage monitoring and maintaining adequate vitamin D levels in pregnancy. While the results highlight a potential link between maternal vitamin D deficiency and some adverse pregnancy outcomes, a well‐designed randomized controlled trial is needed to determine whether vitamin D supplementation during pregnancy will reduce these risks.

## Author Contributions

**Yakubu Ibrahim:** conceptualization, investigation, methodology, writing – original draft, writing – review and editing, data curation, formal analysis, software. **Su Peng Loh:** conceptualization, methodology, supervision, writing – review and editing, funding acquisition. **Nurul Iftida Basri:** conceptualization, methodology, resources, writing – review and editing, supervision, project administration, funding acquisition. **Norshariza Nordin:** conceptualization, methodology, supervision, writing – review and editing. **Amilia Afzan Mohd Jamil:** conceptualization, supervision, writing – review and editing, funding acquisition, methodology.

## Funding

This study has received funding from the Ministry of Higher Education Malaysia, under the Fundamental Research Grant Scheme (FRGS/1/2022/SKK01/UPM/02/1). The funder has no role in the design, data collection, analysis, decision to publish, and preparation of this manuscript.

## Ethics Statement

Approval to conduct the study was obtained from the Universiti Putra Malaysia Research and Ethics Committee involving human subjects (JKEUPM) on the June 28, 2022, with approval number (JKEUPM‐2021‐915). The study was carried out in accordance with the standards of human experimentation, as described in the Helsinki Declaration of 1975, as revised in 2000. Written informed consent was obtained from all eligible participants before enrolment.

## Consent

The authors have nothing to report.

## Conflicts of Interest

The authors declare no conflicts of interest.

## Supporting information


**File S1:** fsn371377‐sup‐0001‐Supplementary file 1.pdf.

## Data Availability

Data from this study is available with the corresponding author upon reasonable request.
