# Supplementary material for: Maternal Vitamin D Deficiency and Its Effects on Pregnancy Outcomes
Source: Food Sci Nutr. 2026 Jan 26;14(1):e71377. doi: 10.1002/fsn3.71377 (PMC12835557; doi:10.1002/fsn3.71377)
Supplement: Supplementary file 1 — File S1: fsn371377‐sup‐0001‐Supplementary file 1.pdf. [file FSN3-14-e71377-s001.pdf]

ID | | | | |

Type of Food or Drink		Name of brand/product	Never or less than 1 per month	1 per Month	2-3 per Month	1 per Week	2 per Week	3-4 per Week	5-6 per Week	1 per Day	2+ per Day	Medium serving	Your Serving Size S	M	L
Fresh milk: any flavor	Full cream											1 cup (8 oz or 250 mL)			
	Low fat														
	Skimmed														
Sterilized milk: any flavor	Full cream											1 cup (8 oz or 250 mL)			
	Low fat														
	Skimmed														
UHT milk: any flavor	Full cream											1 cup (8 oz or 250 mL)			
	Low fat														
	Skimmed														
Milk powder: any flavor	Full cream											3 tablespoons (30g)			
	Low fat														
	Skimmed														
Prenatal milk powder												3 tablespoons (30g)			
Milk in coffee or tea												1 tablespoon			
Milk on cereal (if not included above)												½ cup			
Milk shake												1 cup (8 oz or 250 mL)			
Ice cream: chocolate, vanilla												½ cup			
Soy milk												1 cup (8 oz or 250 mL)			
Evaporated creamer in coffee, tea or others												3 teaspoons (20g)			
Condensed milk in coffee, tea or others												1 teaspoons (20g)			

ID

Type of Food or Drink	Name of brand/product	Never or less than 1 per month	1 per Month	2-3 per Month	1 per Week	2 per Week	3-4 per Week	5-6 per Week	1 per Day	2+ per Day	Medium serving	Your Serving Size S	M	L
Powdered malt drinks: any flavor											3 tablespoons (30g) or 1 sachet (32g)			
Yogurt (regular or soy; frozen)											½ cup (125g, 1 container)			
Cheese											1 slice (21g)			
Breakfast cereal											1 bowl (30g)			
Instant cereal drinks											1 sachet (28g)			
Sandwich bread											2 slices			
Waffle, pancake, French toast											1 piece (1/4 waffle, 4” round)			
Butter (in any foods eaten)											1 pat; teaspoon			
Margarine (in any foods eaten)											1 pat; teaspoon(5g)			
Biscuit (please specified the type of biscuit)											2 to 3 pieces* (20g) (*depends the type of biscuit)			
Macaroni with cheese											1 cup			
Canned salmon											2 tablespoons or 1 cup salmon casserole			
Canned tuna											2 tablespoons or 1 cup tuna casserole			
Canned sardines											2 fish (1/2 can)			
Salmon steak											90 g (3 oz)			
Other fish: white											90 g (3 oz)			
Other fish: oily											90 g (3 oz)			
Cream soups made with milk											1 cup (250 mL)			

ID | | | |

Type of Food or Drink		Name of brand/product	Never or less than 1 per month	1 per Month	2-3 per Month	1 per Week	2 per Week	3-4 per Week	5-6 per Week	1 per Day	2+ per Day	Medium serving	Your Serving Size S	M	L
Eggs: eaten alone or in other foods	Yolk only											1 large egg			
	White only														
	Whole														
Potatoes; mashed with milk and margarine												½ cup (1 scoop)			
Shellfish:mussels												½ cup			
Meat: (please specify the part of meat)															
Beef	with fat											85g (3oz)			
	without fat														
Chicken	with skin											½ chicken breast or 1 drumstick or 85g (3oz)			
	without skin														
Pork												85g (3oz)			
Internal organ: (please specify animal of respective organ)															
Internal organ	Liver											28g (1oz)			
	Kidney														
	Spleen														
	Brain														
